# Conformation Controls Mobility: 2H‐Tetranaphthylporphyrins on Cu(111)

**DOI:** 10.1002/cphc.201901135

**Published:** 2020-02-17

**Authors:** Jan Kuliga, Stephen Massicot, Rajan Adhikari, Michael Ruppel, Norbert Jux, Hans‐Peter Steinrück, Hubertus Marbach

**Affiliations:** ^1^ Lehrstuhl für Physikalische Chemie II Universität Erlangen-Nürnberg Egerlandstr. 3 91058 Erlangen Germany; ^2^ Interdisciplinary Center for Molecular Materials (ICMM) Universität Erlangen-Nürnberg Henkestr. 42 91054 Erlangen Germany; ^3^ Lehrstuhl für Organische Chemie II Universität Erlangen-Nürnberg Henkestr. 42 91054 Erlangen Germany

**Keywords:** chirality, diffusion behavior, porphyrinoids, scanning tunneling microscopy, surface chemistry

## Abstract

The adsorption behavior and the mobility of 2H‐Tetranaphthylporphyrin (2HTNP) on Cu(111) was investigated by scanning tunneling microscopy (STM) at room temperature (RT). The molecules adsorb, like the structurally related 2HTPP, in the “inverted” structure with the naphthyl plane restricted to an orientation parallel to the Cu surface. The orientation of the four naphthyl groups yields altogether 16 possible conformations. Due to the existence of rotamer pairs, 10 different appearances are expected on the surface, and all of them are identified by STM at RT. Most interestingly, the orientation of the naphthyl groups significantly influences the diffusion behavior of the molecules on Cu(111). We identify three different groups of conformers, which are either immobile, medium or fast diffusing at RT. The mobility seems to decrease with increasing size of the footprint of the conformers on the surface.

## Graphical abstract

**Mobile or not**? 2H‐Tetranaphthylporphyrin adsorbs as ten different conformers on Cu(111) at RT. The orientation of the naphthyl groups controls the diffusion behaviour of the molecules, from immobile to medium and fast.

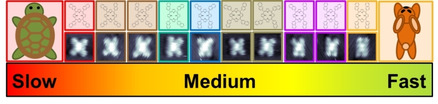

## 1 Introduction

The design and construction of functional architectures on the nanoscale is a very active research topic due to the growing demand for the miniaturization of devices.^[1]^ A most promising approach is the bottom‐up route, where complex organic molecules adsorb and eventually self‐assemble on well‐defined surfaces.^[2]^ The choice of functional groups and molecular as well as substrate symmetry have a major impact on the adsorption behavior of the organic building blocks, and can even induce surface chirality.[^3^, ^4^, ^5^, ^6^, ^7^, ^8^] In particular, the mobility of the molecules on the surface is of great importance, as it is a prerequisite for the formation of molecular networks. Different functional groups, orientation to the surface and shape can contribute to the diffusion behavior and mobility of large organic molecules.[^9^, ^10^, ^11^] Identifying the correlation of specific functional groups with this surface mobility thus is of very high interest.

Porphyrins are a most promising class of functional organic molecules, since they combine an intrinsic functionality governed by the coordination of different metal atoms at the center of the macrocycle, with their conformational flexibility. The latter leads to a large variety of supramolecular structures on surfaces.[^12^, ^13^, ^14^] Scanning tunneling microscopy (STM) has been proven as a powerful tool to investigate such arrangements, their intramolecular conformation and their electronic structure.[^13^, ^15^] Extensive studies^[2]^ have shown that the conformation and supramolecular arrangement strongly depend on the substrate,[^16^, ^17^] molecular coverage,^[18]^ presence and nature of a coordinated metal atom,[^19^, ^20^] heat treatments[^13^, ^21^] and functional side groups.^[22]^ In this regard, 2H‐tetraphenylporphyrin (2HTPP) has become a well‐understood reference system.[^23^, ^24^, ^25^, ^26^, ^27^] On Cu(111), the strong chemical interaction between the iminic nitrogen atoms and the Cu substrate atoms leads to a peculiar intramolecular conformation, the so called “inverted” structure.[^28^, ^29^] Based on the detailed knowledge of this structure, we herein now tackle the changes that occur when replacing the phenyl group by a naphthyl group to introduce a rotation axis in the molecular plane. For 2HTNP on Cu(111), assuming flat adsorption of the naphthyl groups, 16 different surface conformations are expected, including chiral and non‐chiral species. Our systematic study by STM at room temperature documents that these different adsorption conformers can indeed be observed and, most interestingly, that their surface mobility strongly depends on the specific conformation.

## 2 Results and Discussion

**Figure 1:**
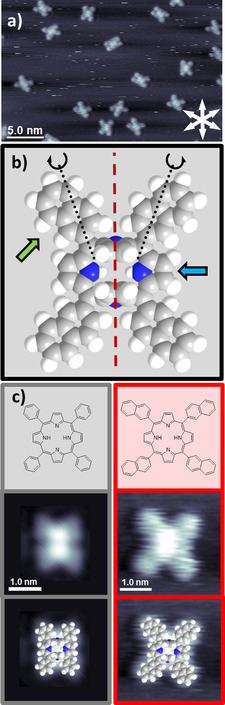
a) Constant‐current STM image of a low coverage of 2HTNP, prepared and measured at RT. b) Top view of a space‐filling model of the “inverted” structure of 2HTNP: Each naphthyl substituent (indicated by a green arrow) can be oriented away (*α*) or towards (*τ*) the main axis of the molecule through the iminic pyrrole groups (dashed red line); also shown is the tilt angle of the iminic (=N−) pyrrole group; the aminic pyrrole group is indicated by the blue arrow. c) Chemical structure, high‐resolution STM image, and structural overlay of inverted space‐filling models of 2HTPP (left) and 2HTNP (right) for comparison.

As a first step, we describe and discuss the general adsorption behavior of 2HTNP on Cu(111). All STM images shown in this section were acquired at RT. Figure 1a depicts an STM image for a coverage of 0.0012 ML (1 ML refers to the one adsorbate molecule per substrate atom); this coverage corresponds to ∼2–3 % of a closed first layer of flat lying molecules. 2HTNPs adsorb as isolated molecules, which are aligned along one of the three main crystallographic directions of Cu(111). High resolution STM images of individual molecules exhibit two (sometimes merged to one) elongated parallel protrusions in the center of the molecule, which are surrounded by four dimmer protrusions in the periphery. This adsorption behavior is very similar to the well‐investigated 2HTPP, which adsorbs as isolated molecules on Cu(111) at low to medium coverages.^[23]^ This specific molecular appearance is due to strong molecule‐substrate interactions via the iminic nitrogen atoms of the porphyrin macrocycle. As a result, the iminic pyrrole groups are rotated slightly more than 90° out of the macrocycle plane, that is, their plane is oriented almost perpendicular to the surface. This structure is referred to as “inverted” and exhibits two protrusions, which are aligned along one of the three main crystallographic directions of Cu(111) (Figure 1c).^[28]^ The strong similarity of the adsorption behavior and appearance of 2HTNP on Cu(111) to that of 2HTPP gives sufficient evidence to safely conclude that 2HTNP also adsorbs in the “inverted” structure. The four dimmer protrusions per molecule in Figure 1a resemble the flat lying naphthyl groups.

A molecular model of the “inverted” 2HTNP is depicted in Figure 1b. The iminic pyrrole groups are rotated upwards, while the aminic pyrrole groups (blue arrow) are parallel to the surface. For 2HTPP, the phenyl groups are parallel to the surface as well, and rotation of the phenyl group around the linking C−C bond by 180° leaves the symmetry of the molecule unchanged; see Figure 1c. In contrast, for 2HTNP, rotation of the flat‐lying naphthyl groups (green arrow) around the linking C−C‐bond (black dotted line) by 180° changes the symmetry of the molecule, which leads to a variety of possible conformations, including surface chiral and non surface chiral ones. In order to describe them, we introduce the *α/τ* ‐notation. In the *α*‐orientation, the naphthyl group points away from the main molecular axis through the iminic pyrrole groups (red dashed vertical line in Figure 1b), while in the *τ‐*orientation it points towards this axis. By indicating the orientations of the four groups clockwise, starting in the left upper corner of the molecule, we obtain 16 possible conformations.

**Figure 2:**
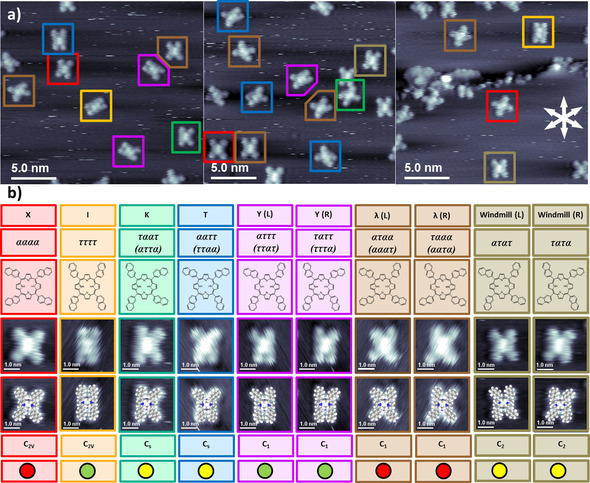
a) Three constant‐current STM images showing the 10 surface conformations of 2HTNP highlighted by differently colored boxes. b) Name, *α/τ* ‐notation, chemical structure, high resolution STM image of a single molecule, STM image overlaid by a scaled space‐filling model, point group, and mobility ranking (red: immobile, yellow: medium, green: fast) for all possible conformations. In case of equivalent rotamers, two *α/τ* ‐notations are denoted.

**Figure 3:**
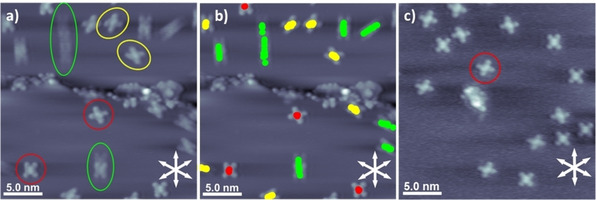
a) Average frames of STM movies of 2HTNP on Cu(111) at RT; *U*=−1.15 V, *I*=30 pA. b) Same image with the positions of the molecules color‐coded in each single STM image, according to the observed mobility (red: immobile, yellow: medium, green: fast), and then superimposed over the corresponding STM average frames. c) Average frames of STM movies of 2HTNP on Cu(111) measured at RT after annealing to 400 K for 1 h; all molecules are immobile, as exemplarily indicated for one molecules; *U*=−1.10 V, *I*=30 pA.

Figure 2a shows three STM images, which contain altogether 10 different conformations of 2HTNP on Cu(111). A common feature is that the molecules are orientated with their main axis along one of the three main symmetry‐equivalent crystallographic substrate directions. The fact that we did not observe 16 different conformations can be explained, if we consider that some molecules are rotamer pairs, that is, rotation by 180° does not lead to a new appearance (see below). In such cases, one appearance has to be attributed to two different *α/τ*‐notations, which are obtained by switching the first two labels with the last two, e. g., *ααττ* equals *τταα* (see Figure 3). This observation also indicates that the C_3v_ symmetry of the substrate can be neglected in the point group considerations of the different adsorbed conformers below.

In the following, all observed conformations are discussed according to their appearance, *α/τ*‐notation, symmetry elements and point group. Selected STM images along with the categorization of the observed conformations are depicted in Figure 2. Starting with the X‐shape conformation, the elongated central protrusion is surrounded by four dimmer protrusions in a symmetrical fashion. The molecule almost forms a square, with the apparent length and width values of 2.12 and 2.19 nm, respectively. According to the *α/τ*‐notation, the conformation is *αααα*. The molecule with two mirror planes belongs to the C_2v_ point group. For the I‐shape, the central protrusion is again surrounded in a symmetric way by four dimmer protrusions, but it displays a rectangular shape with apparent length and width values of 2.25 and 1.48 nm, respectively. The conformation is *ττττ*, and the point group is again C_2V_.

The next two shapes, K and T, are a mixture of the first two (X and I), and belong to the C_s_ point group. The K‐shape has one mirror plane perpendicular to the main axis of the molecule. The two protrusions on the left side resemble the elongated protrusions of the I‐shape, while the two protrusions on the right side mimic the X‐shape. The notations of the corresponding rotamer conformations are *ταατ* and *αττα* . For the T‐shape, the mirror plane is along the molecular axis. It has the two upper protrusions oriented like in the X‐shape and the two lower ones are like in the I‐shape (or vice versa). The notations of the corresponding rotamers are *ααττ* and *τταα*.

All conformations discussed so far are achiral adsorption motives. The remaining conformations are surface chiral, that is, they represent three enantiomeric pairs. In order to simplify the description, only one conformation of the pair is described and for its enantiomeric counterpart only the *α/τ*‐notation is given. The first enantiomeric pair is the windmill shapes L and R, where all naphthyl‐groups have the alternating *α/τ* orientation. Thereby, the *ατατ* conformer has a counterclockwise orientation (L) and the *τατα* conformer a clockwise orientation of the naphthyl groups (R). Both belong to the C_2_ point group with the rotation axis in the center of the macrocycle.

The Y‐shape is very similar to the I‐shape, with one of the dimmer protrusions pointing away from the main molecular axis. The naphthyl‐groups are either orientated *ατττ* (L) or *ταττ* (R) for this enantiomeric pair, or *ττατ* or *τττα* for their rotamers, respectively. The opposite of the Y‐shape is the *λ*‐shape, in which three naphthyl‐groups are pointing away from the main molecular axis and only one protrusion is aligned towards the main molecular axis. This corresponds to *αταα* (L) and *τααα* (R) conformers, with *ααατ* and *αατα* being the corresponding rotamers, respectively. The Y‐shape and the *λ*‐shape both have no symmetry element, and thus belong to the C_1_ point group.

When evaluating the data and searching for the different conformers, it was very difficult to find the windmill conformers, while the rest was observed quite frequently. It is also important to note that we never observed a switching of the orientations of the naphthyl groups and thus a change in molecular conformation at RT. Therefore, we conclude that the observed conformations are stable at RT.

Next, we address the dynamic behavior of the different 2HTNP conformers. For 2HTPP, it is known that it readily diffuses along the main crystallographic axis at RT.^[30]^ Herein, we therefore investigate the influence of the naphthyl groups compared to the phenyl groups, and the impact of their orientation on their mobility. By evaluating STM movies (see Figure S1 in the Supporting Information) and motion‐pathway‐plots (superposition of subsequently measured STM images), we find that the diffusion behavior of the different 2HTNP conformers is quite different. This is evident from Figure 3a and 3b, where motion‐pathway‐plots within a given timeframe are shown. The ten conformers could be classified into three groups, which are indicated in different colors in Figure 3 and also Figure 2 (bottom row). Immobile conformers are indicated in red, conformers with medium mobility in yellow and fast diffusing conformers in green. The X‐ and both *λ*‐shapes both are immobile; they are practically confined to their position and are hardly moving at all. For the K‐, T‐ and both windmill shapes, the mobility is slightly larger and the molecules diffuse short distances along a crystallographic axis in the time interval of the superimposed STM images. Y‐ and I‐conformers are very mobile and diffuse the longest distances along a crystallographic axis.

From these observations, it is obvious that the intramolecular conformation of adsorbed 2HTNP significantly influence the mobility, that is, its diffusion behavior. The detailed analysis of the different conformations shows a systematics behind our observations. Conformations with 4 (I) or 3 (Y) naphthyl groups in *τ‐*orientation, that is, molecules with the smallest footprint, have a higher mobility and diffuse larger distances (green). Molecules with 2 naphthyl groups in *τ‐*orientation (K, T, windmill) behave intermediate (yellow). Finally, conformations with only 1 (λ) or 0 (X) naphthyl groups in *τ‐*orientation, that is, molecules with the largest footprint, are immobile (red). As one possible explanation, one could imagine that for the molecules with all naphthyl groups in *α‐*orientation, that is, for the larger footprint, the interaction potential has a larger corrugation, and thus the whole molecule is immobilized. Interestingly, chirality seems not play any role, as chiral conformers belong to all 3 mobility categories.

As a final step, the behavior of the molecules was investigated after annealing at 400 K for 1 h. Thereafter, only three of the 10 conformations are observed. The X‐shape is the by far dominant conformer, and rarely λ(L)‐ or λ(R)‐shape enantiomers were observed (Figure 3c). Notably, these conformers show no mobility before and after the annealing step. From its lack of mobility and the fact that the other molecules are converted to this conformation upon providing thermal energy, we conclude that the X‐shape is the thermodynamically most favorable conformation, i. e. poses an energetic minimum.

## 3 Conclusions

In summary, we observe a very peculiar adsorption behavior of 2HTNP on Cu(111). At RT, individual molecules are observed by high‐resolution STM, which are orientated along the main crystallographic directions. The molecules have an inverted structure with upstanding iminic pyrrole groups, similar to 2HTPP on the same substrate. Due to the asymmetry of the naphthyl groups, 16 different surface conformers are possible, 10 of which have a different appearance in STM. The complete set could indeed be identified. While all conformers are stable at RT, the investigation of their diffusional behavior shows different mobilities, which can be classified into three categories, that is, low, medium and high. Interestingly, the mobility is related to the naphthyl orientation relative to the main axis of the molecule along the iminic nitrogen atoms. Fast diffusing species have 3 or 4 naphthyl groups pointing in the direction of the movement (aligned with the crystallographic axis), immobile species have only 0 or 1 naphthyl groups orientated in this direction (which equals 4 or 3 groups oriented away from the main molecular axis). This behavior shows that the introduction of asymmetric functional groups in the periphery linked to the macrocycle via single C−C bonds can have a strong influence on the adsorption behavior by yielding a variety of non‐chiral and chiral conformers, which behave differently in their diffusion. This is an important observation, which could be used to tailor the diffusion properties, e. g., by introduction of rigid bonds to the macrocycle or groups that cannot rotate due to steric hindering.

## Conflict of interest

The authors declare no conflict of interest.

## Supporting information

As a service to our authors and readers, this journal provides supporting information supplied by the authors. Such materials are peer reviewed and may be re‐organized for online delivery, but are not copy‐edited or typeset. Technical support issues arising from supporting information (other than missing files) should be addressed to the authors.

Supplementary
