# Supplementary material for: Conformation Controls Mobility: 2H‐Tetranaphthylporphyrins on Cu(111)
Source: Chemphyschem. 2020 Feb 17;21(5):423–7. doi: 10.1002/cphc.201901135 (PMC7687165; doi:10.1002/cphc.201901135)
Supplement: Supplementary file 1 — Supplementary [file CPHC-21-423-s001.pdf]

CHEMPHYSCHEM

Supporting Information

Conformation Controls Mobility: 2H-Tetranaphthylporphyrins on Cu(111)

Jan Kuliga, Stephen Massicot, Rajan Adhikari, Michael Ruppel, Norbert Jux, Hans-Peter Steinrück, and Hubertus Marbach*© 2019 The Authors. Published by Wiley-VCH Verlag GmbH & Co. KGaA. This is an open access article under the terms of the Creative Commons Attribution License, which permits use, distribution and reproduction in any medium, provided the original work is properly cited.

Supporting information

Experimental section

The experiments and sample preparations were performed in a two-chamber ultrahigh vacuum (UHV) system at a background pressure in the low 10^{-10} mbar regime. The variable temperature scanning tunneling microscope (STM) is an RHK UHV VT STM 300 with RHK SPM 1000 electronics. All STM images were acquired at room temperature (RT) in constant current mode with a Pt/Ir tip and the bias was applied to the sample. The STM images were processed with WSxM software[1] and moderate filtering (Gaussian smoothing, background subtraction) was applied for noise reduction. The preparation of the clean Cu(111) surface was done by repeated cycles of Ar^+ sputtering (600 eV) and annealing to 850 K. The 2HTNP molecules were deposited onto the metal substrates held at RT, by thermal sublimation from a home-built Knudsen cell at 350 °C.

Tunneling parameters for the STM images shown in the manuscript:

Figure 1: a) U_{bias} = -1.15 V, I_{set} = 30 pA; c) U_{bias} = -1.40 V, I_{set} = 37 pA

Figure 2: a-c) U_{bias} = -1.15 V, I_{set} = 30 pA

Figure 3: a,b) U_{bias} = -1.15 V, I_{set} = 30 pA; c) U_{bias} = -1.10 V, I_{set} = 30 pA

STM movies were acquired to study the diffusion behavior of the 2HTNP. Figure S1 shows nine consecutive STM frames of such a movie. The frames were used to create a motion-pathway-plot by marking each individual molecule with a dot and superimposing all frames on top of each other. The center of the macrocycle with the two inverted pyrrole groups was used as a center of the dot. According to their mobility three different colors were used to emphasize the different mobility of the conformers. Green resembles fast, yellow medium diffusing molecules and red static conformers.

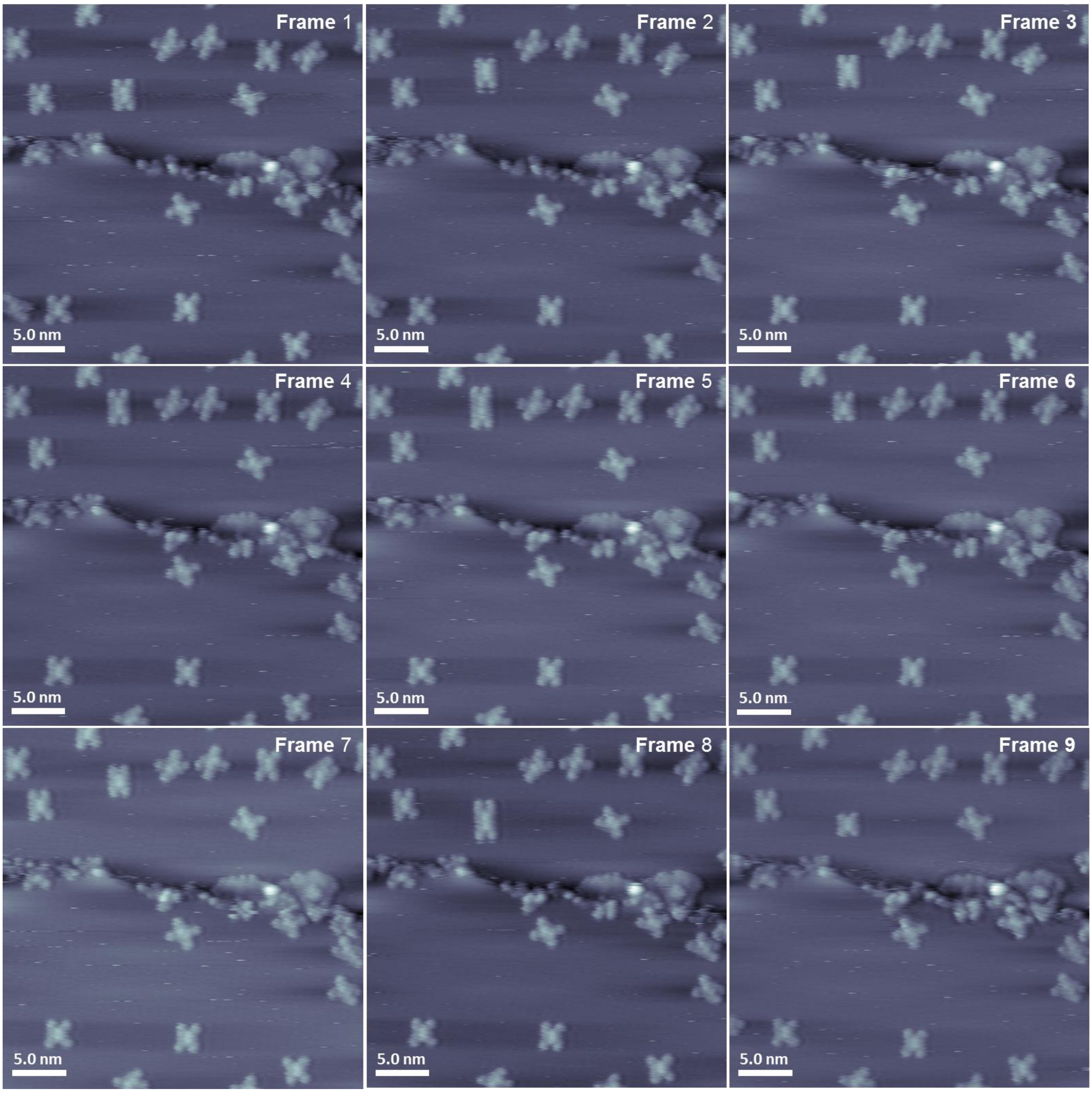

Figure S1: *Subsequent STM images taken to acquire an STM movie and to create a motion-pathway-plots of 2HNTP.*

Synthesis

All chemicals were purchased from Sigma-Aldrich®, Acros Organics®, Fluka®, Fisher Scientific® or Alfa Aesar® and used without further purification. Pyrrole and solvents for chromatography were distilled prior to usage. Dichloromethane was distilled from K_2CO_3. Thin layer chromatography (TLC) was performed on Merck silica gel 60 F524, detected by UV-light (254nm, 366nm). Column chromatography was performed on Macherey-Nagel silica gel 60 M (230-400 mesh, 0.04–0.063 mm). NMR spectroscopy was performed on a Bruker Avance 400 (^{1}H: 400 MHz, ^{13}C: 100 MHz). Deuterated solvents were purchased from

Sigma Aldrich and used as received. ^{1}H NMR and ^{13}C NMR chemical shifts δ are given in parts per million [ppm] and are referenced to residual protic impurities in the solvent ($CHDCl_2$: ^{1}H: 5.32 ppm), or to the deuterated solvent itself (CD_2Cl_2: ^{13}C: 53.8 ppm). The resonance multiplicities are indicated as “s“ (singlet), “d” (doublet), “t” (triplet), “q” (quartet) and “m” (multiplet). Signals referred to as “bs” (broad singlet) are not clearly resolved or significantly broadened. LDI/MALDI-ToF (nitrogen UV-laser, 337 nm) mass spectra were obtained by using a Bruker ultrafleXreme spectrometer with 2,5-dihydroxybenzoic acid (DHB) or (*E*)-2-(3-(4-(*tert*-butyl)phenyl)-2-methylallylidene)malononitrile (dctb) as matrices. ESI/APPI-ToF mass spectrometry was carried out on a Bruker maXis 4G UHR TOF MS/MS-spectrometer or a Bruker micrOTOF II focus TOF MS-spectrometer. UV/vis spectroscopy was carried out on a Varian Cary 5000 UV-Vis-NIR spectrometer. Spectra were recorded at room temperature using quartz cuvettes with a path length of 1 cm. Fluorescence spectra were recorded on a Shimadzu RF–5301PC spectrofluorophotometer.

2*H*-tetrakis-(β-naphthyl)-porphyrin (**2HTNP**)

Pyrrole (692 µL, 671 mg, 10.0 mmol) and β-naphthylaldehyde (1.56 g, 10.0 mmol) were dissolved in CH_2Cl_2 (1 L), and degassed by passing nitrogen through the solution for 15 min. Then BF_3 diehtyl etherate (407 µL, 468 mg, 3.30 mmol) was added, and the mixture was stirred under light exclusion for 2 h at room temperature. After the addition of DDQ (1.70 g, 7.50 mmol), the mixture was stirred for additional 2 h at room temperature. The solvent was removed, and the resulting residue was filtered over silica gel (CH_2Cl_2, diameter: 4.0 cm, length: 15 cm). The purple band was collected and concentrated. Precipitation with methanol yielding the product as purple solid.

Yields: 24% (493 mg, 605 µmol).

^{1}H NMR (400 MHz, CDCl3, rt): δ [ppm] = 8.89 (s, 8H), 8.70 (bs, 4H), 8.41 (d, 4H, ^{3}J = 8.1 Hz), 8.21–8.17 (m, 8H), 8.10–8.08 (m, 4H), 7.73–7.68 (m, 8H), -2.59 (bs, 2H).

^{13}C NMR (100 MHz, CDCl3, rt): δ [ppm] = 139.9, 134.1, 133.2, 133.0, 132.6, 131.8, 128.8, 128.3, 127.3, 127.0, 126.2, 120.6.

HRMS (APPI): m/z calc. for $C_{60}H_{38}N_4$ [M^+]: 814.309099; found: 814.308261.

UV/Vis (CH_2Cl_2 + 1% NEt_3) λ [nm] (ε [$M^{-1}cm^{-1}$]) 424 (29000), 462 (513000), 518 (20000), 555 (11100), 593 (6500), 649 (4900).

Fluorescence (CH_2Cl_2 + 1% NEt_3, λ_{exc} = 424 nm) λ [nm] 655, 718.

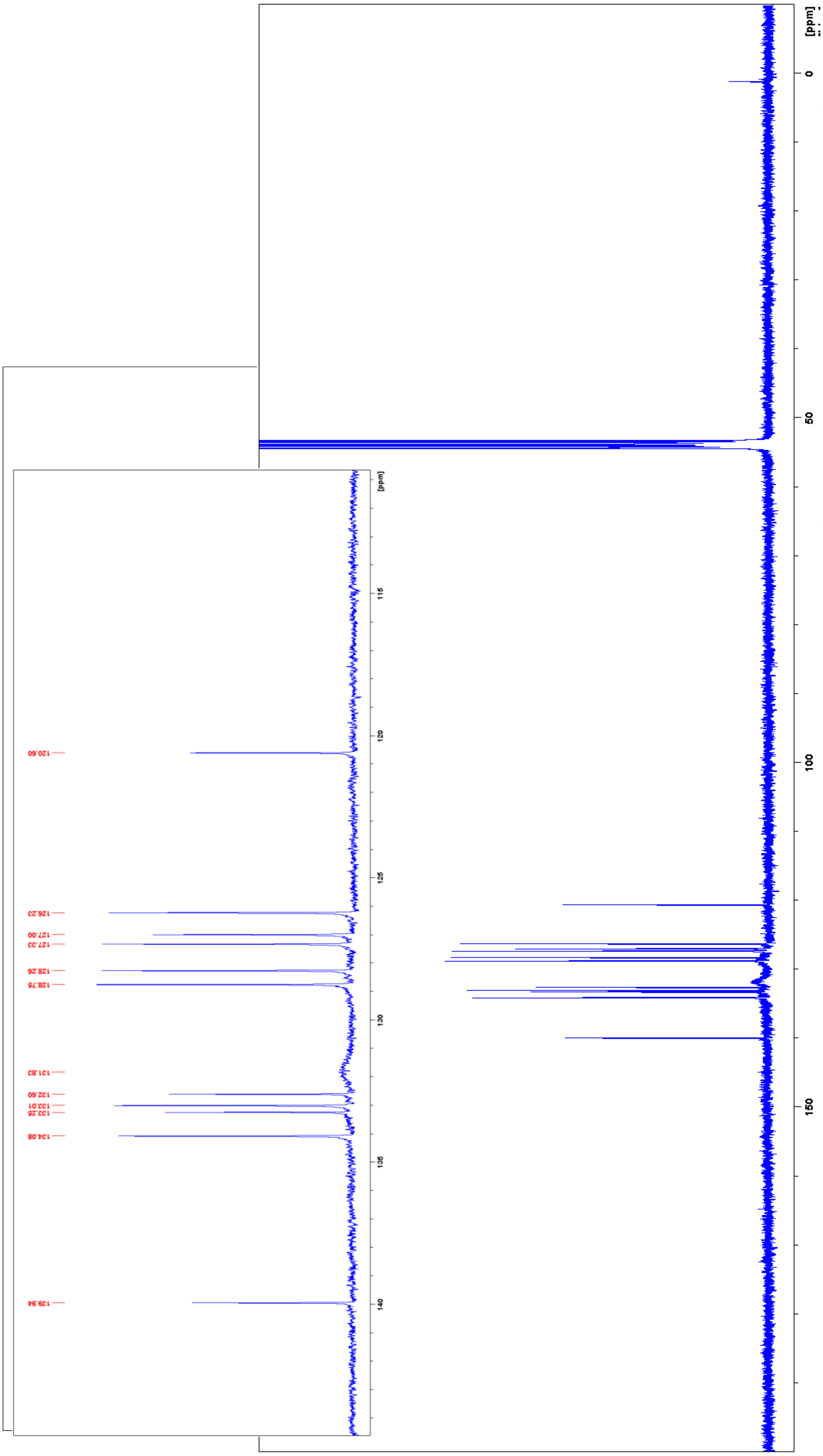

<u>FFigure S3:</u> ^{13}C NMR of **2HTNP**.

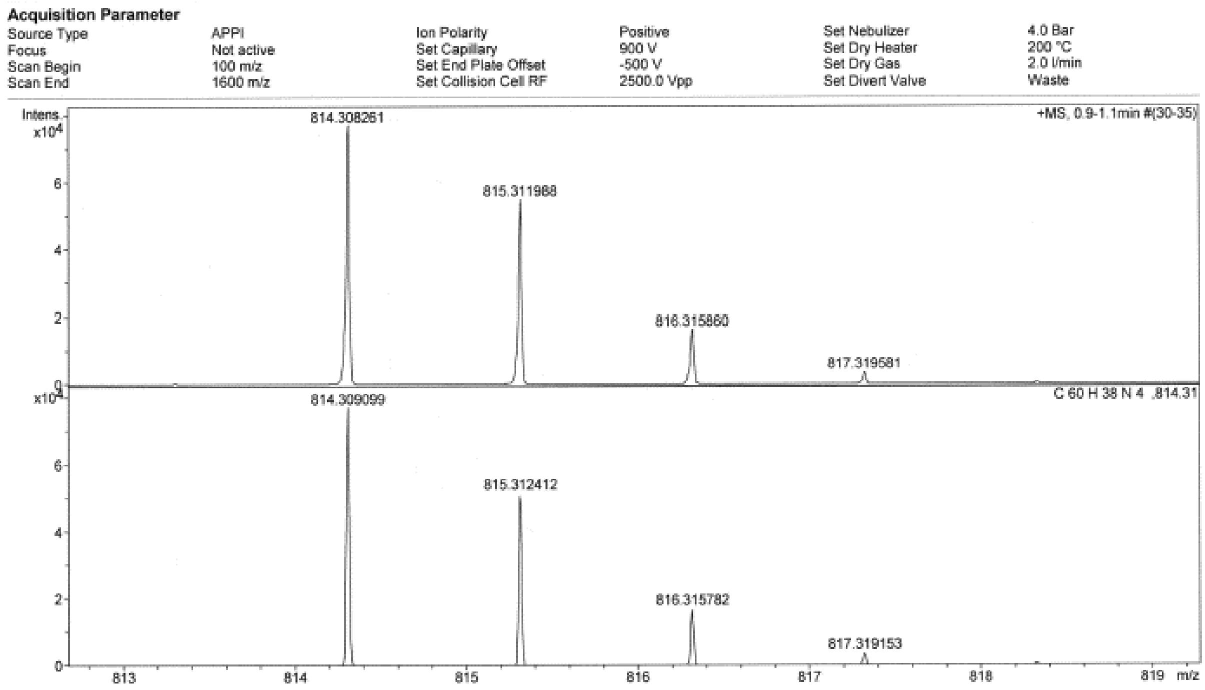

Figure S4: *HRMS (APPI, toluene) of* ***2HTNP****.*

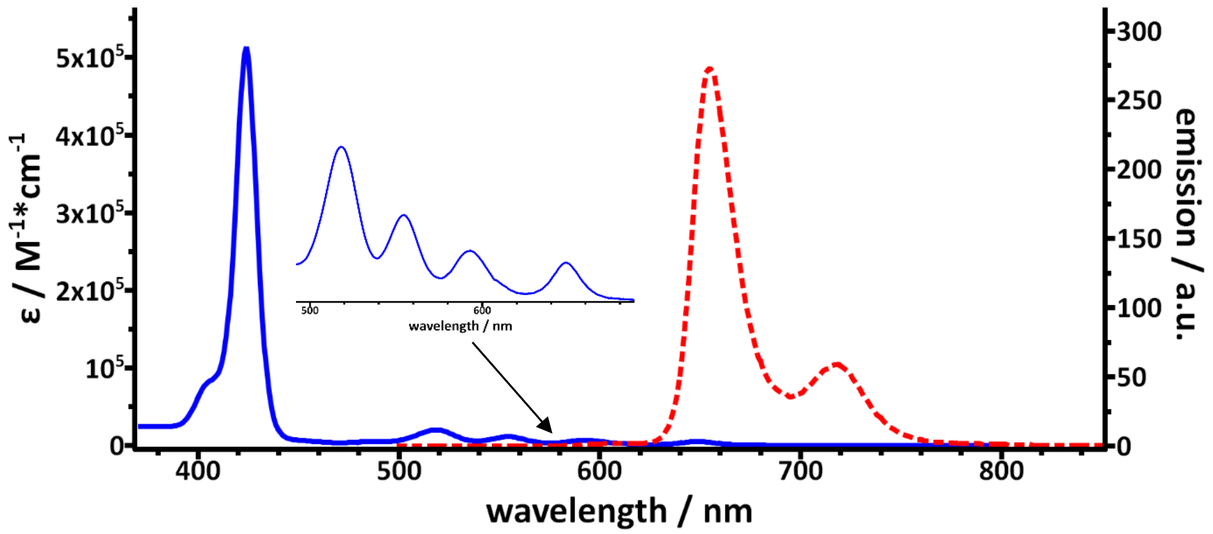

Figure S5: *Absorption (blue line) and emission spectrum of* ***2HTNP*** *(dashed red line; excitation at 424 nm) measured in* CH_2Cl_2 *+ 1%* NEt_3 *at RT.*

References

1. I. Horcas, R. Fernández, J. Gomez-Rodriguez, J. Colchero, J. Gómez-Herrero and A. Baro, *Rev. Sci. Instrum.* **2007**, 78, 013705.
